# Inhibition of Trigeminal Nociception by Non-invasive Vagus Nerve Stimulation: Investigating the Role of GABAergic and Serotonergic Pathways in a Model of Episodic Migraine

**DOI:** 10.3389/fneur.2020.00146

**Published:** 2020-03-05

**Authors:** Lauren E. Cornelison, Sara E. Woodman, Paul L. Durham

**Affiliations:** Center for Biomedical and Life Sciences, Missouri State University, Springfield, MO, United States

**Keywords:** neck inflammation, pain, sensitization, sumatriptan, nitric oxide, vagus nerve

## Abstract

Migraine is a prevalent neurological disease that is characterized by unpredictable episodic attacks of intense head pain. The underlying pathology involves sensitization and activation of the trigeminal system. Although non-invasive vagus nerve stimulation (nVNS) is recommended for the treatment of migraine, the abortive mechanism of action is not well-understood. The goal of this study was to compare the ability of nVNS and sumatriptan to inhibit trigeminal activation in two animal models of episodic migraine and to investigate the receptor mechanism of action of nVNS. Nocifensive head withdrawal response was investigated in adult male Sprague Dawley rats using von Frey filaments. To induce trigeminal nociceptor sensitization, complete Freund's adjuvant was injected in the trapezius muscle and trigeminal neurons were activated by exposure to a pungent odor or injection of the nitric oxide donor sodium nitroprusside. Some animals received nVNS or sumatriptan as treatment. Some animals were injected intracisternally with antagonists of GABA_A_, 5-HT3 or 5-HT7 receptors prior to nVNS since these receptors are implicated in descending modulation. While unsensitized animals exposed to the pungent odor or nitric oxide alone did not exhibit enhanced mechanical nociception, sensitized animals with neck muscle inflammation displayed increased trigeminal nocifensive responses. The enhanced nociceptive response to both stimuli was attenuated by nVNS and sumatriptan. Administration of antagonists of GABA_A_, 5-HT3, and 5-HT7 receptors in the upper spinal cord suppressed the anti-nocifensive effect of nVNS. Our findings suggest that nVNS inhibits trigeminal activation to a similar degree as sumatriptan in episodic migraine models via involvement of GABAergic and serotonergic signaling to enhance central descending pain modulation.

## Highlights

- Neck muscle inflammation mediated sensitization of the trigeminal system to a pungent odor or nitric oxide that promoted mechanical nociception.
- nVNS inhibited trigeminal nociception in two models of episodic migraine.
- The inhibitory effects of nVNS involve GABAergic and serotonergic pathways.

## Introduction

Migraine is a prevalent neurological disease characterized by unpredictable episodic attacks of severe head pain that is accompanied by autonomic symptoms including photophobia, phonophobia, and nausea (1). The disease burden of migraine is significant since it disproportionally affects women of childbearing age and negatively impacts performance at school and work, and interferes with family and social activities (2–4). Migraine pathology involves sensitization and activation of the trigeminal system, which provides sensory innervation to much of the head and face including the meninges (5). Recently, non-invasive electrical stimulation of the vagus nerve has been reported to be beneficial in the treatment of migraine and cluster headache (6–9). The pathological pain associated with migraine involves activation of trigeminal ganglion nerves, which provide sensory innervation of the head and face and relay nociceptive signals to the spinal trigeminal nucleus (STN) (10). The use of a non-invasive vagus nerve stimulator (nVNS, gammaCore™) is FDA approved for the acute (episodic) and preventive (episodic and chronic) treatment of cluster headache and the acute treatment of migraine in adult patients. Additionally, results from clinical trials have provided evidence that nVNS is a safe and well-tolerated therapeutic option (6, 9). Importantly, the reported 2-h pain-free rate for nVNS in treating episodic migraine is similar to that of the triptans (11). Thus, nVNS is proposed as a novel non-pharmacological therapeutic alternative or complement to the triptan class of abortive migraine drugs. Although similarly effective to triptans, nVNS likely functions via different physiological and cellular mechanisms to modulate pain signaling in response to trigeminal nerve activation. The mechanism by which triptans function to block trigeminal pain is thought to involve inhibiting the release of calcitonin-gene related peptide (CGRP) and other pro-inflammatory molecules from peripheral and central terminals of the trigeminal nerve as well from the cell body within the ganglion (12). In contrast, the inhibitory effect of nVNS as an acute migraine treatment is proposed to promote multiple distinct cellular changes and pathways within the brain and spinal cord to facilitate descending pain modulation (13). The descending inhibitory pathway is known to involve activation of 5-HT3 and 5-HT7 receptors on inhibitory interneurons that stimulates release of glycine and GABA, which act as inhibitory neurotransmitters of primary or secondary trigeminal nociceptors (14). Thus, the reported efficacy of nVNS may involve modulation of GABAergic and serotonergic signaling but this pathway has not been demonstrated in episodic migraine models.

Migraineurs are genetically predisposed to development of a hyperexcitable nervous system that is susceptible to multiple risk factors, which function to promote peripheral and central sensitization or can act as triggers to initiate a migraine attack (15). Premonitory symptoms may include increased sensitivity to physical stimuli such as flickering lights, loud, or irregular sounds, or even pungent odors such as those from the California bay laurel (CBL) or headache tree (16, 17). Similar to other complex neurological diseases, stress and anxiety are reported migraine risk factors that can significantly influence disease onset, progression, and maintenance of the clinical phenotype (18) and can manifest as increased tension and pain in neck and shoulder muscles (19). Chronic muscle tension and inflammation in the neck and shoulders can mediate persistent muscle fiber contraction, local ischemia, and the release of pro-inflammatory mediators that facilitate sensitization of primary and secondary nociceptors (20). The convergence in the upper spinal cord of nerves providing sensory innervation of neck/shoulder muscles and those emanating from the trigeminal ganglion may explain why neck/shoulder pathology is often cited as a risk factor for orofacial pain conditions including migraine (21, 22). In support of this notion, neck muscle inflammation has been reported to promote sensitization of primary trigeminal neurons so that exposure to a known migraine trigger, the pungent odor from a CBL leaf extract, was sufficient to cause an increase in trigeminal nociception in response to mechanical stimulation (23). One of the main active molecules in CBL trees leaves is umbellulone, which has been shown to cause activation of TRPA1, the subsequent release of CGRP, and to increase trigeminal nociception (17). Another factor known to promote activation of trigeminal nociceptors in animal models of migraine is nitric oxide (24). Using nitric oxide donors to mimic migraine pathophysiology is supported by human data that infusion of a nitric oxide donor in migraine susceptible individuals will trigger a migraine attack (25). Thus, a goal of this study was to compare the efficacy of nVNS to sumatriptan in two animal models of episodic migraine involving trigeminal sensitization mediated by neck muscle inflammation and trigeminal activation via either a pungent odor or nitric oxide. Another goal was to investigate the mechanism of action of nVNS to inhibit trigeminal nociception.

## Methods

### Animals

One hundred and ninety-five adult (d45-d56) Sprague Dawley male rats (200–300 g), were purchased from Missouri State University's Central Management Breeding Colony (Springfield, MO) and allowed to acclimate for 1 week to facility conditions prior to use. Animals were housed individually in plastic rat cages with aspen chip bedding and unrestricted access to both food and water in a room with 12 h light/dark cycles. All protocols were approved by Missouri State University's Institutional Animal Care and Use Committee and conducted in compliance with the Animal Welfare Act, National Institutes of Health, and ARRIVE Guidelines. Concerted efforts were made to minimize suffering, as well as the number of animals. The attending veterinarian provided guidance on appropriate dosing of all compounds and also determined if animals were to be removed from the study due to excessive suffering. One hundred eighty-two animals were used for final analysis, due to exclusion of outliers that were defined as average values more than 2 standard deviations from the mean of that group at one or more timepoints. No animals were removed from the study due to ill health.

### Sensitization and Activation of Trigeminal Nociceptive Neurons

The experimental design for the first episodic migraine model was based on a prior study and involves activation of sensitized trigeminal neurons in response to exposure to a pungent odor (23). Animals were placed under 3% isoflurane and received 10 injections of 10 μl of complete Freund's adjuvant (CFA, Sigma-Aldrich, St. Louis, MO; 1:1 in 0.9% sterile saline) into the upper trapezius. Animals were monitored for normal behaviors for a total of 8 days. To cause activation of trigeminal nociceptors, animals were exposed for 10 min to the volatile compounds from an oil extract obtained from California bay laurel tree leaves (CBL, World Spice, Seattle, WA) that was prepared as described previously (23).

In the second episodic migraine model, nitric oxide was used to mediate trigeminal nociceptor activation in sensitized animals 8 days post trapezius CFA injection. Animals were lightly anesthetized using 3% isoflurane and injected intraperitoneally at a dose of 0.01 mg/kg with sodium nitroprusside (SNP, Sigma-Aldrich) dissolved in sterile 0.9% saline. This dose was chosen since it did not cause increased nociception in naïve animals and hence was determined to be subthreshold. In control animals, an equal volume of sterile saline was injected.

### nVNS and Sumatriptan Treatments

The procedure for nVNS was performed essentially as described previously (23). Animals were lightly anesthetized under 3% isoflurane and the stimulator electrodes placed on a shaved area over the vagus nerve. Initially, a 1 ms pulse of 5 kHz sine waves, repeated at 25 Hz, for 2 min was administered that was followed 5 min later by a second 2 min stimulation. Animals receiving sumatriptan were given a dose of 0.3 mg/kg subcutaneously, which was shown previously to effectively inhibit trigeminal activation (26).

### Inhibitor Injections

Animals were lightly anesthetized using 3% isoflurane prior to intracisternal injection of antagonists to GABA_A_ and the 5-HT3 and 5-HT7 receptors. Bicuculline (GABA_A_ inhibitor, Tocris Bioscience, Minneapolis, MN) was dissolved in DMSO, then diluted to 20 μM in sterile 0.9% saline, while Ondansetron Hydrochloride (5-HT3 inhibitor, Tocris) and SB 269970 (5-HT7 inhibitor, Tocris) were dissolved in 0.9% sterile saline to a final concentration of 100 nM. In addition, a mixture of 100 nM Ondansetron and 100 nM SB 269970 was prepared in sterile saline. All inhibitors were administered via injection of 20 μl between the occipital bone and C1 vertebrae to naïve animals or delivered immediately prior to nVNS (2 h post odor exposure). Bicuculline was also administered to sensitized animals that received CBL exposure with no nVNS treatment.

### Nocifensive Behavior Testing

Behavioral changes were the primary outcome measured in this study. Changes in nocifensive response to mechanical stimulation of trigeminal neurons were determined essentially as described (23). Prior to nociception testing, animals were allowed to acclimate to the Durham Animal Holder (UGO Basile, Gemonio, Italy) in the designated procedure room for 5 min on 3 consecutive days. To minimize reflexive or startle responses, animals were conditioned to a mechanical stimulus by gently rubbing the hair in the facial region with a pipette tip. This method measures deep musculoskeletal pain responses rather than cutaneous, reflexive defensive responses and hence higher weight filaments were required to test nociception. Following acclimations, animals were allowed to rest for 48 h prior to baseline assessments.

Mechanical nocifensive thresholds were determined in response to a series of calibrated von Frey filaments (Stoelting, Wood Dale, IL) between 8 a.m. and 12 p.m. Nocifensive withdrawal reactions, defined as a head withdrawal observed prior to the bending of the filament, were verified by two scientists blinded to the experimental conditions. Each filament was applied 5 times over both the right and left areas of each animal and reported as an average number of reactions. Animals were randomly sorted into groups, and baseline measurements were established prior to any treatments. Animals that responded on average more than 2.5 times to the 100 g filament during baseline measurements were not included in the study. Additional measurements were taken 8 days post-muscle injections, 2 h post odor exposure or SNP injections, 1 h post nVNS or sumatriptan treatments, and 24 h post treatment. Animals were euthanized following testing via CO_2_ asphyxiation and decapitation.

### Statistical Design and Analysis

An a priori power analysis using G^*^Power Software (Dusseldorf, Germany), allowing for comparison between groups at 5 time points, resulted in a recommended minimum of 5 animals per group to detect effects of treatments. Following collection, data were evaluated for normality using a Shapiro–Wilk test. Behavioral data were found to be non-normal (*P* < 0.05), so non-parametric statistical tests were applied. To determine if nociception was different across all groups, a Kruskal–Wallis test was performed. Upon reaching a significant result, a Mann–Whitney *U*-test with a Wilcoxon's W *post-hoc* test was performed to determine if there were pairwise differences in nociception between groups at each evaluated time point. Statistical analysis was performed using SPSS Statistical Software 24 (IBM), and changes were considered significant if *P* < 0.05.

## Results

**Figure 1:**
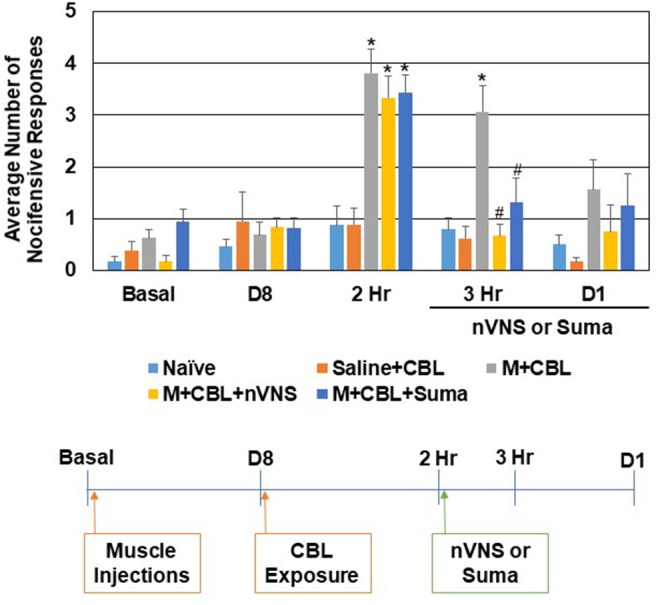
nVNS and sumatriptan inhibit trigeminal nociception mediated by a pungent odor in sensitized animals. The average nocifensive head withdrawal response ±SEM of 5 applications to each side to mechanical stimuli are reported. Some animals were left untreated (Naïve), some were injected with saline in the trapezius (Saline) and exposed to the pungent extract from California Bay Laurel leaves (CBL), while others were injected with complete Freund's adjuvant 8 days prior to exposure to CBL (M+CBL) and nociceptive responses determined at 2 h, 3 h, and 1 day post CBL exposure. Some of the M+CBL animals were treated with nVNS or sumatriptan and nociception measured 1 h and 1 day post treatment. *P < 0.05 when compared to Naïve while #*P* < 0.05 compared to M+CBL.

Initially, the level of trigeminal nociception to mechanical stimulation was determined with the use of von Frey filaments in a model of episodic migraine (Figure 1). The average number of nocifensive head withdrawals to mechanical stimulation was <1 response out of 5 applications at the basal time point for all experimental conditions. At day 8, the nociceptive response for all conditions including animals that received upper trapezius injection of CFA were similar to basal levels. In sensitized animals mediated by neck muscle inflammation however (*n* = 8), the average number of nocifensive responses was significantly (*P* < 0.05) elevated over naïve (*n* = 12) levels 2 h after exposure to the pungent odor from a CBL extract (*P* < 0.001) but not in animals injected with saline (*n* = 9) in the upper trapezius (*P* = 0.39). One hour after treatment with nVNS (*n* = 7) or sumatriptan (*n* = 8) (3 h after odor exposure) a significant decrease (*P* = 0.001, *P* = 0.028) in nociception was observed when compared to untreated sensitized animals, which were still elevated at this time point (*P* < 0.001). The average number of nocifensive responses was no longer significantly different between any groups 1 day post odor exposure or treatment with nVNS or sumatriptan (*P* = 0.071). No change in nociception was observed in animals receiving only saline at 3 h and day 1 (*P* = 0.62, *P* = 0.89).

**Figure 2:**
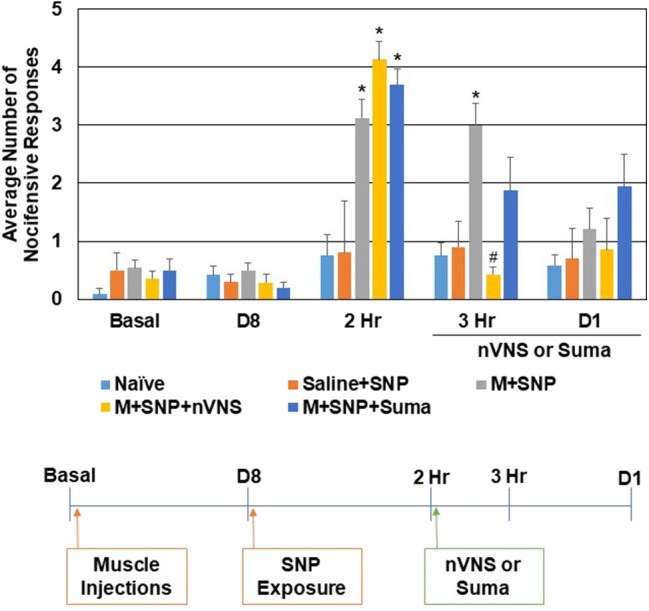
nVNS and sumatriptan inhibit trigeminal nociception mediated by nitric oxide in sensitized animals. The average nocifensive head withdrawal response ±SEM of 5 applications to each side to mechanical stimuli are reported. Some animals were left untreated (Naïve), some were injected with saline in the trapezius (Saline) and exposed to the nitric oxide donor sodium nitroprusside (SNP), while others were injected with complete Freund's adjuvant 8 days prior to exposure to SNP (M+SNP) and nociceptive responses determined at 2 h, 3 h, and 1 day post SNP exposure. Some of the M+SNP animals were treated with nVNS or sumatriptan and nociception measured 1 h and 1 day post treatment. *P < 0.05 when compared to Naïve while #*P* < 0.05 compared to M+SNP.

The effect of nVNS and sumatriptan were also compared in a second animal model of episodic migraine. In this model, sensitization of trigeminal nociceptive neurons was mediated by injection of CFA in the upper trapezius 8 days prior to injection of the nitric oxide donor sodium nitroprusside (SNP), which was used to trigger activation and a nocifensive response (Figure 2). Consistent with the CBL data, in sensitized animals mediated by neck muscle inflammation, the average number of nocifensive responses was significantly (*P* < 0.05) elevated over naive levels 2 h after injection of SNP (*n* = 12, *P* < 0.001) but not in animals injected with saline in the upper trapezius prior to SNP injection (*n* = 6, *P* = 0.61). One hour after treatment with nVNS (3 h after CBL) a significant decrease (*n* = 7, *P* < 0.001) in nociception was observed when compared to untreated sensitized animals, which were still significantly elevated over naive (*P* < 0.001). Sumatriptan also caused a decrease in the average number of withdrawal responses such that the response was not significantly different from SNP-stimulated animals or naive levels (*n* = 8, *P* = 0.13, *P* = 0.15). The average number of nocifensive responses was no longer significantly different between groups 1 day post SNP or treatment with nVNS or sumatriptan (*P* = 0.15). No change in nociception was observed in animals receiving only saline at 3 h and day 1 (*P* = 0.89, *P* = 0.96).

**Figure 3:**
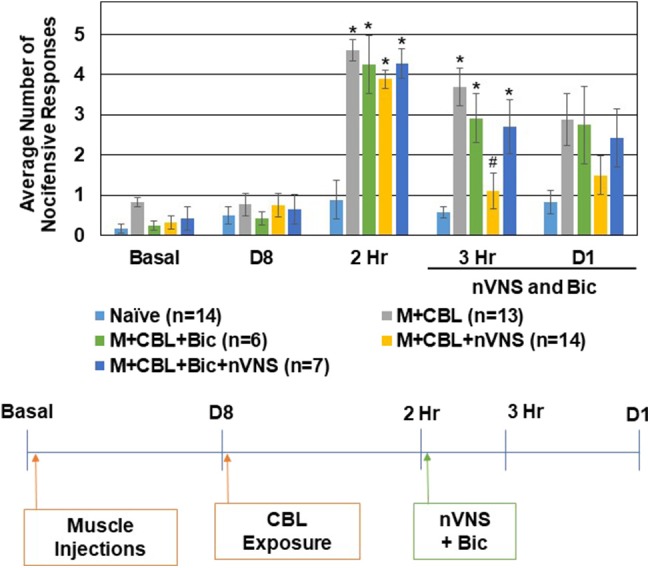
Inhibitory effect of nVNS on trigeminal nociception involves GABA_A_. The average nocifensive head withdrawal response ±SEM of 5 applications to each side to mechanical stimuli are reported. Some animals were left untreated (Naïve), some were injected with complete Freund's adjuvant 8 days prior to exposure to a pungent extract from California Bay Laurel leaves (M+CBL) and nociceptive responses determined at 2 h, 3 h, and 1 day post CBL exposure. Some of the M+CBL animals were treated with Bicuculline and/or nVNS 2 h after CBL exposure and nociception measured 1 h and 1 day post treatment. *P < 0.05 when compared to Naïve while #*P* < 0.05 compared to M+CBL.

To determine if intracisternal administration of inhibitors of the GABA_A_, 5-HT3, and 5-HT7 receptors would cause a change in the basal level of trigeminal nociception to mechanical stimulation, unsensitized animals received injections of selective antagonists and nocifensive responses were measured at the same time points as the episodic migraine models. Administration of the GABA_A_ inhibitor Bicuculline (20 μM) or a mixture of antagonists of 5-HT3 (Ondansetron, 100 nM) and 5-HT7 (SB-269970, 100 nM) did not mediate a significant difference in the average number of nocifensive responses at any of the time points (data not shown). To test if the inhibitory effect of nVNS on trigeminal nociception observed in the CBL odor-induced episodic migraine model involved GABA_A_ signaling, the GABA_A_ receptor antagonist was administered just prior to nVNS. All of the animals exhibited a similar level of nocifensive response to mechanical stimulation at the day 8 time point prior to CBL exposure (Figure 3). As before, sensitized animals exposed to CBL (*n* = 13) exhibited elevated nocifensive responses at 2 h when compared to naïve animals (*n* = 14) (M + CBL, *P* < 0.001; M + CBL + nVNS, *P* = 0.001; M + CBL + Bic + nVNS, *P* = 0.003). As expected, nVNS (*n* = 14) significantly inhibited (*P* < 0.05) the level of nociception mediated by CBL in sensitized animals 1 h post treatment (*P* = 0.002). Administration of the GABA_A_ receptor antagonist Bicuculline (20 μM) prior to nVNS, however, suppressed the inhibitory effect of nVNS, which resulted in the average number of nocifensive responses being significantly different from Naïve levels (*n* = 7, *P* = 0.004). However, animals treated with Bicuculline prior to nVNS were not significantly elevated compared to animals receiving nVNS alone (*P* = 0.067). As a control, Bicuculline administered to sensitized animals immediately following CBL exposure did not potentiate or inhibit the nocifensive response and was significantly elevated when compared to Naïve levels (*n* = 6, *P* = 0.001). At day 1 post treatments, no significant differences in trigeminal nociception were observed although the trends were similar to the 3 h time point.

**Figure 4:**
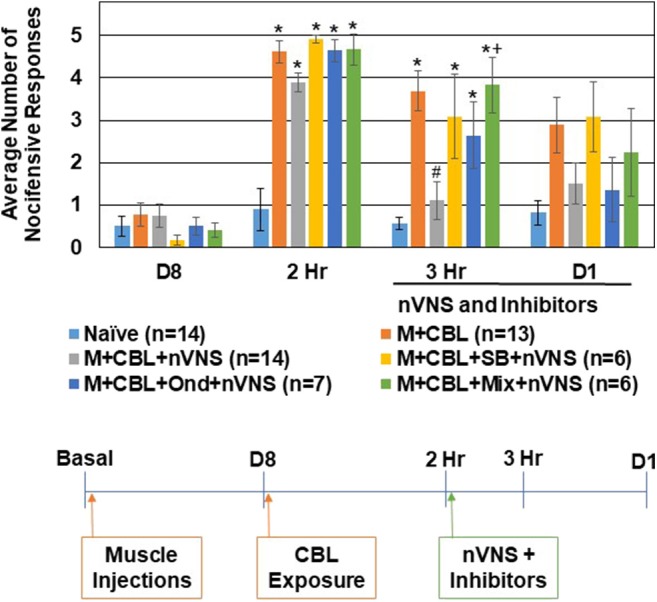
Anti-nociceptive effect of nVNS involves 5-HT3 and 5-HT7 receptors. The average nocifensive head withdrawal response ±SEM of 5 applications to each side to mechanical stimuli are reported. Some animals were left untreated (Naïve), some were injected with complete Freund's adjuvant 8 days prior to exposure to a pungent extract from California Bay Laurel leaves (M+CBL) and nociceptive responses determined at 2 h, 3 h, and 1 day post CBL exposure. Some of the M+CBL animals were treated with Ondansetron, SB 269970, or a mixture (Mix) prior to nVNS and nociception measured 1 h and 1 day post treatment. *P < 0.05 when compared to Naïve while #*P* < 0.05 compared to M+CBL. +*P* < 0.05 when compared to M + CBL + nVNS.

To determine if the inhibitory effect of nVNS on trigeminal nociception observed in the CBL odor-induced episodic migraine model also involved activation of 5-HT receptors, selective 5-HT3 and 5-HT7 antagonists were injected intracisternally prior to nVNS. All of the animals exhibited a similar level of nocifensive response to mechanical stimulation at the day 8 time point prior to CBL odor exposure (Figure 4). In this experiment, all sensitized animals exhibited a robust increase (*P* < 0.05) in the average number of nocifensive responses following pungent odor exposure for each experimental condition. While nVNS significantly inhibited (*P* = 0.002) the level of nociception mediated by CBL odor in sensitized animals 1 h post treatment, administration of the 5-HT3 antagonist Ondansetron (100 nM) (*n* = 7), 5-HT7 antagonist SB 269970 (100 nM) (*n* = 6), or a mixture (100 nM of each) (*n* = 6), prior to nVNS suppressed the inhibitory effect of nVNS. The average number of nocifensive responses for animals treated with Ondansetron, SB 269970, or the mixture was significantly different from naïve levels (*P* = 0.011, *P* = 0.05, *P* < 0.001, respectively). Animals treated with Ondansetron or SB 269970 prior to nVNS were not significantly elevated compared to animals receiving nVNS alone (*P* = 0.064, *P* = 0.108, respectively). However, animals treated with the mixture were significantly elevated from M + CBL + nVNS animals (*P* = 0.007). At day 1 post treatments, no significant differences in trigeminal nociception were observed when compared to naïve levels.

## Discussion

The major finding from our study was that nVNS was as effective as sumatriptan in inhibiting trigeminal nociception in two different rodent models of episodic migraine. In both models, sensitization of trigeminal neurons was mediated by neck muscle inflammation, which is a reported migraine risk factor (27, 28). In this primed state, exposure of the animals to the pungent odor from a CBL extract or a nitric oxide donor was sufficient to cause a significant transient increase in trigeminal nociception to mechanical stimulation. Exposure to either triggering agent in unsensitized animals, however, did not result in an enhanced state of trigeminal nociception. In this way, these models are designed to mimic pathophysiological events associated with episodic migraine in humans. Importantly, nVNS and sumatriptan were both effective in inhibiting the increased level of trigeminal nociception mediated by CBL odor and nitric oxide in sensitized animals. This finding is consistent with human studies that have reported nVNS provides a therapeutic benefit that is similar to that of sumatriptan for the acute treatment of episodic migraine (11). Our finding that nVNS inhibits the average number of nocifensive responses to mechanical stimulation mediated by a pungent odor is also consistent with results from an earlier study (23) and with results from other animal studies that mimic aspects of migraine pathology (24, 29, 30) and other types of orofacial pain (31, 32). While previous studies have utilized nitric oxide donors to directly cause trigeminal nociception (24, 33, 34), in our model, a subthreshold concentration of sodium nitroprusside promoted activation of trigeminal nocifensive response in sensitized animals. In this way our model is designed to mimic human studies in which nitric oxide infusion causes a migraine attack in migraine susceptible individuals (25). An interesting feature of our model is that trigeminal nociception is not elevated by upper trapezius inflammation but rather a sensitized or primed state of nociceptors is promoted. This pathological condition mimics a commonly cited risk factor since neck muscle pain and tenderness are reported during the prodrome and attack phases of migraine (27, 28). Neck muscle inflammation is likely to mediate central sensitization of the trigeminal system via increased peripheral signaling since afferent projections from these muscles terminate in the upper spinal cord and subsequently converge with the trigeminal system (22, 35). This supports the notion that neck muscle inflammation could promote central sensitization by activating ascending nociceptive second order neurons or by facilitating downregulation or dysregulation of the inhibitory descending pain modulation pathway. These events would result in an increase in the allostatic load and promote development of a hypersensitive or hyperexcitable state of the trigeminal system that would be more responsive to inflammatory stimuli, which is characteristic of migraine pathophysiology (36).

Although nVNS and sumatriptan are reported to have similar efficacy in treating episodic migraine, the pathways by which each of these abortive therapies function to inhibit trigeminal pain signaling are likely to be mediated via different cellular and molecular mechanisms. Based on animal studies, the inhibitory effects of sumatriptan are thought to be primarily mediated via direct modulation of primary trigeminal neurons and involve blocking the release of CGRP and the excitatory neurotransmitter glutamate (12). Hence, sumatriptan's mechanism of action would inhibit neurogenic inflammation in the dura, inhibit neuron-glia communication in the ganglion, and also inhibit activation of second order neurons and glia cells within the spinal cord to decrease peripheral and central sensitization of the trigeminal system. In contrast, the primary effects of nVNS are likely to be multimodal and would involve modulation of central cellular activities that regulate descending pain inhibition pathways (37). The findings from our study provide evidence for the involvement of GABA_A_ receptors and 5-HT3 and 5-HT7 receptors in mediating the inhibitory effect of nVNS in an episodic migraine model. Specifically, intracisternal injection of the GABA_A_ receptor antagonist Bicuculline or administration of the 5-HT_7_ receptor antagonist SB 269970 and the 5-HT_3_ receptor antagonist Ondansetron, or a mixture of the two antagonists, significantly inhibited nVNS repression of trigeminal nociception. The inhibitory effect of nVNS is likely to be mediated by activation of GABA_A_ receptors on primary and second order neurons (32, 38, 39), which would result in neuronal hyperpolarization via an influx of chloride to inhibit neurotransmitter release. Although not a focus of this study, the source of 5-HT is likely from activation of descending projections from the rostroventromedial medulla (RVM), which functions as a final relay in the control of descending pain facilitation. The activation of 5-HT_3_ and 5-HT_7_ receptors on inhibitory neurons by nVNS would enhance the descending inhibitory pain pathway via activation of spinal interneurons and release of the inhibitory neurotransmitters, GABA and glycine, to suppress ascending pain transmission. This mechanism is supported by other orofacial pain studies involving trigeminal nerve activation in which direct stimulation of the vagus nerve was shown to exhibit anti-nociceptive effects, to facilitate the serotonergic descending inhibition pathway, and to modulate inhibition of GABAergic neurons (14, 40, 41). Other mechanisms may also be involved in nVNS inhibition of trigeminal pain signaling. In a previous study (23), nVNS treatment of sensitized animals inhibited CBL odor-stimulated nuclear expression of the signaling protein P-ERK in trigeminal ganglia. In the same model of episodic migraine utilized in this study, nVNS also inhibited stimulated expression of GFAP and Iba1, which are biomarkers of activated astrocytes and microglia, respectively (42). These findings are suggestive that nVNS can inhibit cellular changes implicated in peripheral and central sensitization. nVNS has also been reported to inhibit the nitroglycerin-mediated increase in glutamate levels in cerebral spinal fluid in a model of trigeminal allodynia (24). Another possible mechanism of nVNS involves the direct regulation of pain signaling in the upper spinal cord based on data from a recent human imaging study that provided evidence of the trigeminal and vagus systems being interconnected at the level of the spinal trigeminal nucleus (43). Taken together, the inhibitory effect of nVNS in migraine is facilitated via multiple mechanisms that function to suppress peripheral and central sensitization of the trigeminal system and inhibit pain signaling.

In summary, exposure to a pungent odor or administration of nitric oxide, which are both reported migraine triggers in humans, resulted in an enhanced nocifensive state in response to mechanical stimulation of trigeminal neurons in animals with ongoing neck muscle inflammation, another reported risk factor associated with migraine pathology. nVNS was as effective in inhibited trigeminal nociception as sumatriptan in two rodent models of episodic migraine. We propose that the inhibitory effect of nVNS is mediated, in part, via activation of 5-HT3 and 5-HT7 receptors on inhibitory neurons within the spinal trigeminal nucleus that results in release of GABA and subsequent activation of GABAA receptors on sensory neurons. However, 5-HT released from the RVM could also directly modulate sensory neurons via activation of other serotonergic receptors. Given its central mechanism of action involving GABAergic and serotonergic pathways associated with descending pain modulation, nVNS offers a non-pharmacological alternative or adjunctive therapy to triptans.

## Data Availability Statement

The datasets generated for this study are available on request to the corresponding author.

## Ethics Statement

The animal study was reviewed and approved by IACUC Missouri State University.

## Author Contributions

LC was primarily responsible for the collection and analysis of the behavioral results and assisted in the writing and editing of the final manuscript. SW was primarily responsible for the inhibitor studies and assisted in drafting the original manuscript and editing of final version. PD was responsible for the study design, directing research efforts, and final analysis of the results as well as writing and editing of the manuscript.

### Conflict of Interest

This work was supported by a research grant from electroCore, Inc. The funder had the following involvement with the study: study design, decision to publish, and assistance in editing of the manuscript.

## References

[1] Headache Classification Committee of the International Headache Society (IHS) The International Classification of Headache Disorders 3rd ed. Cephalalgia. (2018) 38:629–808. 10.1177/0333102413485658. 29368949

[2] Burch R Rizzoli P Loder E. The prevalence and impact of migraine and severe headache in the United States: figures and trends from government health studies. Headache. (2018) 58:496–505. 10.1111/head.13281 29527677

[3] Buse DC Scher AI Dodick DW Reed ML Fanning KM Manack Adams A. Impact of migraine on the family: perspectives of people with migraine and their Spouse/domestic partner in the CaMEO Study. Mayo Clin Proc. (2016) 10.1016/j.mayocp.2016.02.013 27132088

[4] Loder S Sheikh HU Loder E. The prevalence, burden, and treatment of severe, frequent, and migraine headaches in US minority populations: statistics from national survey studies. Headache. (2015) 55:214–28. 10.1111/head.12506 25644596

[5] Bernstein C Burstein R. Sensitization of the trigeminovascular pathway: perspective and implications to migraine pathophysiology. J Clin Neurol. (2012) 8:89–99. 10.3988/jcn.2012.8.2.89 22787491 PMC3391624

[6] Goadsby PJ de Coo IF Silver N Tyagi A Ahmed F Gaul C. Non-invasive vagus nerve stimulation for the acute treatment of episodic and chronic cluster headache: a randomized, double-blind, sham-controlled ACT2 study. Cephalalgia. (2018) 38:959–69. 10.1177/0333102417744362 29231763 PMC5896689

[7] de Coo IF Marin JC Silberstein SD Friedman DI Gaul C McClure CK. Differential efficacy of non-invasive vagus nerve stimulation for the acute treatment of episodic and chronic cluster headache: ameta-analysis. Cephalalgia. (2019) 39:967–77. 10.1177/0333102419856607 31246132 PMC6637721

[8] Martelletti P Barbanti P Grazzi L Pierangeli G Rainero I Geppetti P Consistent effects of non-invasive vagus nerve stimulation (nVNS) for the acute treatment of migraine: additional findings from the randomized, sham-controlled, double-blind PRESTO trial. J Headache Pain. (2018) 19:120 10.1186/s10194-018-0949-9 30563446 PMC6755543

[9] Tassorelli C Grazzi L de Tommaso M Pierangeli G Martelletti P Rainero I. Noninvasive vagus nerve stimulation as acute therapy for migraine: the randomized PRESTO study. Neurology. (2018) 91:e364–73. 10.1212/WNL.0000000000005857 29907608 PMC6070381

[10] NosedaRBursteinR. Migraine pathophysiology: anatomy of the trigeminovascular pathway and associated neurological symptoms, cortical spreading depression, sensitization, and modulation of pain. Pain. (2013) 154(Suppl. 1):S44–53. 10.1016/j.pain.2013.07.02123891892

[11] Obermann M Holle D. Recent advances in the management of migraine. F1000Res. (2016) 5:2726. 10.12688/f1000research.9764.1 29098075 PMC5642308

[12] Bigal ME Krymchantowski AV Hargreaves R. The triptans. Expert Rev Neurother. (2009) 9:649–59. 10.1586/ern.09.15 19402776

[13] Henssen D Derks B van Doorn M Verhoogt N Van Cappellen van Walsum AM Staats P. Vagus nerve stimulation for primary headache disorders: an anatomical review to explain a clinical phenomenon. Cephalalgia. (2019) 39:1180–94. 10.1177/0333102419833076 30786731 PMC6643160

[14] Tanimoto T Takeda M Nishikawa T Matsumoto S. The role of 5-hydroxytryptamine3 receptors in the vagal afferent activation-induced inhibition of the first cervical dorsal horn spinal neurons projected from tooth pulp in the rat. J Pharmacol Exp Ther. (2004) 311:803–10. 10.1124/jpet.104.070300 15215286

[15] Aurora SK Wilkinson F. The brain is hyperexcitable in migraine. Cephalalgia. (2007) 27:1442–53. 10.1111/j.1468-2982.2007.01502.x 18034688

[16] Friedman DI de ver Dye T. Migraine and the environment. Headache. (2009) 49:941–52. 10.1111/j.1526-4610.2009.01443.x 19545255

[17] Nassini R Materazzi S Vriens J Prenen J Benemei S de Siena G. The 'headache tree' via umbellulone and TRPA1 activates the trigeminovascular system. Brain. (2012) 135:376–90. 10.1093/brain/awr272 22036959

[18] Buse DC Silberstein SD Manack AN Papapetropoulos S Lipton RB. Psychiatric comorbidities of episodic and chronic migraine. J. neurol. (2013) 260:1960–9. 10.1007/s00415-012-6725-x 23132299

[19] Ohrbach R Dworkin SF. Five-year outcomes in TMD: relationship of changes in pain to changes in physical and psychological variables. Pain. (1998) 74:315–26. 10.1016/S0304-3959(97)00194-2 9520246

[20] Graven-Nielsen T Arendt-Nielsen L. Peripheral and central sensitization in musculoskeletal pain disorders: an experimental approach. Curr Rheumatol Rep. (2002) 4:313–21. 10.1007/s11926-002-0040-y 12126583

[21] Sessle BJ. Neural mechanisms and pathways in craniofacial pain. Can J Neurol Sci. (1999) 26(Suppl. 3):S7–11. 10.1017/S0317167100000135 10563227

[22] Chandler MJ Qin C Yuan Y Foreman RD. Convergence of trigeminal input with visceral and phrenic inputs on primate C1-C2 spinothalamic tract neurons. Brain res. (1999) 829:204–8. 10.1016/S0006-8993(99)01348-7 10350551

[23] Hawkins JL Cornelison LE Blankenship BA Durham PL. Vagus nerve stimulation inhibits trigeminal nociception in a rodent model of episodic migraine. Pain Rep. (2017) 2:e628. 10.1097/PR9.0000000000000628 29392242 PMC5741328

[24] Oshinsky ML Murphy AL Hekierski H Jr Cooper M Simon BJ. Noninvasive vagus nerve stimulation as treatment for trigeminal allodynia. Pain. (2014) 155:1037–42. 10.1016/j.pain.2014.02.009 24530613 PMC4025928

[25] Juhasz G Zsombok T Modos EA Olajos S Jakab B Nemeth J. NO-induced migraine attack: strong increase in plasma calcitonin gene-related peptide (CGRP) concentration and negative correlation with platelet serotonin release. Pain. (2003) 106:461–70. 10.1016/j.pain.2003.09.008 14659530

[26] Buzzi MG Carter WB Shimizu T Heath H III Moskowitz MA. Dihydroergotamine and sumatriptan attenuate levels of CGRP in plasma in rat superior sagittal sinus during electrical stimulation of the trigeminal ganglion. Neuropharmacology. (1991) 30:1193–200. 10.1016/0028-3908(91)90165-8 1663596

[27] Ashina S Bendtsen L Lyngberg AC Lipton RB Hajiyeva N Jensen R. Prevalence of neck pain in migraine and tension-type headache: a population study. Cephalalgia. (2015) 35:211–9. 10.1177/0333102414535110 24853166

[28] Florencio LL Chaves TC Carvalho GF Goncalves MC Casimiro EC Dach F. Neck pain disability is related to the frequency of migraine attacks: a cross-sectional study. Headache. (2014) 54:1203–10. 10.1111/head.12393 24863346

[29] Akerman S Simon B Romero-Reyes M. Vagus nerve stimulation suppresses acute noxious activation of trigeminocervical neurons in animal models of primary headache. Neurobiol Dis. (2017) 102:96–104. 10.1016/j.nbd.2017.03.004 28286178

[30] Chen SP Ay I de Morais AL Qin T Zheng Y Sadeghian H. Vagus nerve stimulation inhibits cortical spreading depression. Pain. (2016) 157:797–805. 10.1097/j.pain.0000000000000437 26645547 PMC4943574

[31] Bossut DF Maixner W. Effects of cardiac vagal afferent electrostimulation on the responses of trigeminal and trigeminothalamic neurons to noxious orofacial stimulation. Pain. (1996) 65:101–9. 10.1016/0304-3959(95)00166-2 8826496

[32] Takeda M Tanimoto T Nishikawa T Ikeda M Yoshida S Ito M. Volume expansion suppresses the tooth-pulp evoked jaw-opening reflex related activity of trigeminal neurons in rats. Brain Res Bull. (2002) 58:83–9. 10.1016/S0361-9230(02)00763-3 12121817

[33] Harris HM Carpenter JM Black JR Smitherman TA Sufka KJ. The effects of repeated nitroglycerin administrations in rats; modeling migraine-related endpoints and chronification. J Neurosci Methods. (2017) 284:63–70. 10.1016/j.jneumeth.2017.04.010 28442295

[34] He W Long T Pan Q Zhang S Zhang Y Zhang D. Microglial NLRP3 inflammasome activation mediates IL-1beta release and contributes to central sensitization in a recurrent nitroglycerin-induced migraine model. J Neuroinflammation. (2019) 16:78. 10.1186/s12974-019-1459-7 30971286 PMC6456991

[35] Morch CD Hu JW Arendt-Nielsen L Sessle BJ. Convergence of cutaneous, musculoskeletal, dural and visceral afferents onto nociceptive neurons in the first cervical dorsal horn. Eur J Neurosci. (2007) 26:142–54. 10.1111/j.1460-9568.2007.05608.x 17614945

[36] Borsook D Maleki N Becerra L McEwen B. Understanding migraine through the lens of maladaptive stress responses: a model disease of allostatic load. Neuron. (2012) 73:219–34. 10.1016/j.neuron.2012.01.001 22284178

[37] Moller M Schroeder CF May A. Vagus nerve stimulation modulates the cranial trigeminal autonomic reflex. Ann neurol. (2018) 84:886–92. 10.1002/ana.25366 30362165

[38] Alhaider AA Lei SZ Wilcox GL. Spinal 5-HT3 receptor-mediated antinociception: possible release of GABA. J Neurosci. (1991) 11:1881–8. 10.1523/JNEUROSCI.11-07-01881.1991 2066767 PMC6575470

[39] Matsumoto S Takeda M Tanimoto T. Effects of electrical stimulation of the tooth pulp and phrenic nerve fibers on C1 spinal neurons in the rat. Exp Brain Res. (1999) 126:351–8. 10.1007/s002210050742 10382620

[40] Tanimoto T Takeda M Matsumoto S. Suppressive effect of vagal afferents on cervical dorsal horn neurons responding to tooth pulp electrical stimulation in the rat. Exp Brain Res. (2002) 145:468–79. 10.1007/s00221-002-1138-1 12172658

[41] Yanes JA. Toward a multimodal framework of brainstem pain-modulation circuits in migraine. J Neurosci. (2019) 39:6035–7. 10.1523/JNEUROSCI.0301-19.2019 31366717 PMC6668207

[42] Dodds KN Beckett EA Evans SF Grace PM Watkins LR Hutchinson MR. Glial contributions to visceral pain: implications for disease etiology and the female predominance of persistent pain. Transl Psychiatry. (2016) 6:e888. 10.1038/tp.2016.168 27622932 PMC5048206

[43] Henssen D Derks B van Doorn M Verhoogt NC Staats P Vissers K. Visualizing the trigeminovagal complex in the human medulla by combining *ex-vivo* ultra-high resolution structural MRI and polarized light imaging microscopy. Sci Rep. (2019) 9:11305. 10.1038/s41598-019-47855-5 31383932 PMC6683146

